# Can regulators have a role in facilitating the development of new medicines for Parkinson's disease?

**DOI:** 10.1177/1877718X251360584

**Published:** 2025-08-06

**Authors:** Mário Miguel Rosa, Ewa Balkowiec-Iskra

**Affiliations:** 1 Lisbon Medical School, Faculdade de Medicina, Universidade de Lisboa, Lisbon, Portugal; 2 Scientific Advice Working Party, EMA, Amsterdam, The Netherlands; 3 Central Nervous System Working Party, EMA, Amsterdam, The Netherlands; 4 Department of Experimental and Clinical Pharmacology, Medical University of Warsaw, Warsaw, Poland; 5 The Office for Registration of Medicinal Products, Medical Devices and Biocidal Products, Warsaw, Poland; 6 Committee for Medicinal Products for Human Use, European Medicines Agency, Amsterdam, The Netherlands

**Keywords:** marketing approval, approval of new drugs, Parkinson's disease, regulatory science, neuropharmacology, regulatory authorities

## Abstract

Parkinson's disease (PD) is the second most frequent neurodegenerative disorder, and its prevalence has doubled in the past 25 years. New formulations of levodopa have recently been marketed that improved the ability to treat PD, but in the European Union, no new active substance for PD has been marketed since 2015, whilst two new agents have been marketed elsewhere. In spite of being the most treatable neurodegenerative disorder, several unmet medical needs still exists in PD, and efforts both from researchers, drug developers and regulators are needed to facilitate further developments in the field. There are several reasons that may account for the lack of new treatments for PD. Regulatory agencies provide scientific advice and have developed several programs to foster drug development. This manuscript discusses the main obstacles identified in the development process, the present status of approvals in European Union and the United States, and the presently available mechanisms to optimize drug development and marketing.

## Plain language summary

Parkinson's disease is a common condition. Although there are several symptomatic treatments available, there are still many unmet medical needs in this disease. Many researchers and drug developers put a lot of efforts in designing and conducting studies aimed to demonstrate efficacy and safety of new compounds, intended for use in patients with Parkinson's disease. Yet, no new active substance for Parkinson's disease has been approved in the European Union since 2015. This article outlines the expected requirements for clinical trials to assess the efficacy and safety of new drugs for the treatment of Parkinson's disease and discusses how regulatory agencies can support investigators to facilitate clinical trials and foster the marketing of efficacious and safe new medicines.

## Introduction

As it is well known, Parkinson's disease (PD), is a neurodegenerative disease characterized by motor (bradykinesia, postural instability, resting tremor and rigidity) and non-motor symptoms (the most frequent being daytime sleepiness, orthostatic hypotension, depression, psychotic symptoms, pain, and sensory disorders).^1^

The global prevalence of PD is around 10 million people worldwide and PD is the second most frequent age-related neurodegenerative disease.^2,3^ Importantly, the prevalence of PD has doubled in the past 25 years.^4^ PD constitutes also a huge financial burden. According to estimates**,** in 2019 PD resulted in 5.8 million disability-adjusted life years and caused 329,000 deaths.^5^

Although research targeting various purported mechanisms of PD, have been ongoing for years, no disease modifying treatment has been approved so far. Up to a certain extent, PD symptoms are controlled through both pharmacological and non-pharmacological methods.

North America and Europe account for about 76% of worldwide prescription medicines market sales.^6^ China and Japan account for 11.8%, and the rest of the World 12.2%. If we consider new medicines launched between 2018–2023, the United States (US) and the European Union (EU) account for over 82% of the market. We will therefore focus on EU and US market. While in US approval is always centralized by the US Food and Drug Administration (FDA), in the EU, new anti-Parkinsonian agents must be centrally (European Medicines Agency, EMA) approved, but older drugs may still be approved on a national basis (e.g., bromocriptine, lisuride, cabergoline, benztropine, trihexyphenidyl), and may thus only be available in some of the countries.

There are several reasons that may account for the lack of new treatments for PD. Existence of several symptomatic treatments with reasonable efficacy and only few promising new potential sites of action may decrease the investigation drive. Moreover, new proposed stagings of disorder as well as new possible biomarkers and the need for improved efficacy assessment tools which require a close discussion among stakeholders may be perceived as an additional burden for the development of new agents for the treatment of PD.

**Figure 1.**
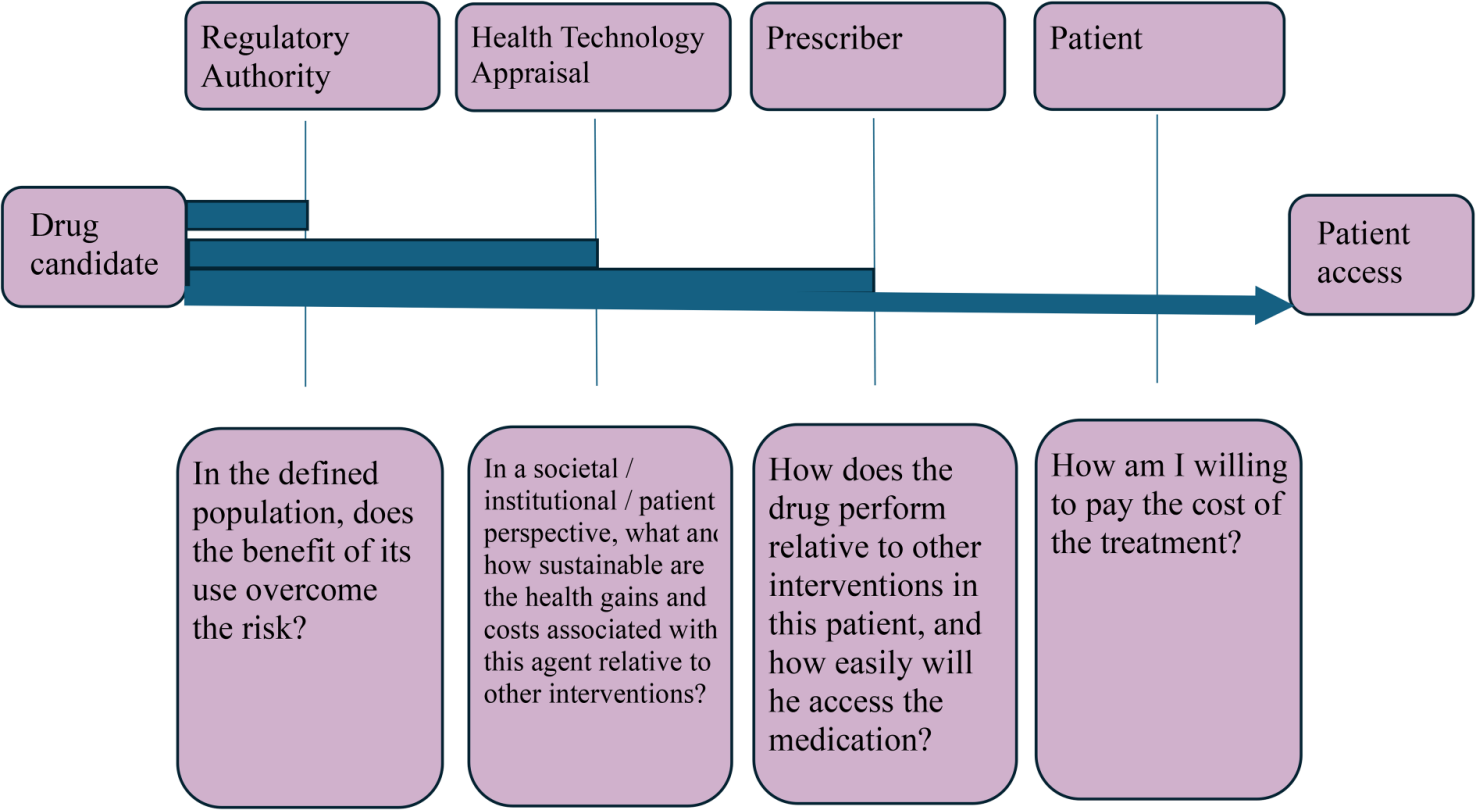
Road to patient access to drugs. Adapted from Eichler et al.^7^

On the other hand, almost all prescription medicines require licensing for medicines to be used in the treatment, and a licensed product has a guarantee of benefit/risk appraisal. This is not the rule with prescription in the setting of open label extensions of clinical trials, via compassionate use or expanded access, and even less so with dietary products. The role of regulators in the medicines development and approval should be well known, but it is acknowledged that this is not the case, and regulators often do not publicize their work, relevant to the scientific community and the society^7^ (Figure 1).

## The approvals and denies in the past 25 years

Of note, a medicinal product may not be marketed in different jurisdictions either because (a) no one has applied for it; (b) someone applied but understood that it would fail approval and withdraws the application; or (c) the product fails approval. Information on the assessment for marketing authorization application (MAA) is usually only available as a résumé if a negative recommendation has been issued and the product MAA has been rejected, or if the product was withdrawn before a negative opinion was issued. Publicly available regulatory information is thus scarce.

Medicinal products approved for the treatment of PD are commonly classified into dopaminergic and non-dopaminergic. Being degeneration of nigrostriatal dopaminergic neurons the hallmark of PD, treatment for patients with PD mostly relies on dopaminomimetic drugs.

The dopaminomimetic drugs include levodopa under several formulations, dopaminergic agonists, monoamine oxidase-B enzyme (MAO-B) inhibitors, and catechol-ortho- methyltransferase (COMT) inhibitors. Nondopaminergic drugs are amantadine and anticholinergics (Table 1).

**Table 1.** Medicines approved for the treatment of PD in the EU and US.

Dopaminergic medicines		
*Dopaminergic agonists*	Approval in the EU	Approval in the US
Pramipexole	Yes	Yes
Ropinirole	Yes	Yes
Apomorphine	Yes	Yes
Rotigotine	Yes	Yes
*MAO-B inhibitors*		
Selegiline	Yes	Yes
Rasagiline	Yes	Yes
Safinamide	Yes	Yes
*COMT inhibitors*		
Entacapone	Yes	Yes
Tolcapone	Yes	Yes
Opicapone	Yes	Yes
Non-dopaminergic medicines		
*NMDA antagonists*		
Amantadine	Yes	Yes
*Anticholinergics*		
Trihexyphenidyl	No	Yes
Benztropine	No	Yes
*Adenosine receptor antagonists*		
Istradefylline	No	Yes

Anticholinergics are old drugs and are thus available via national marketing authorization. In contrast, istradefylline, an adenosine receptor antagonist indicated as adjunctive treatment to levodopa/carbidopa in adult patients with PD experiencing “off” episodes, has been approved in the US in 2019 but is not approved in the EU. The EMA after re-examination in 2021, confirmed its recommendation to refuse MA, since the results of the studies submitted to support a MA were inconsistent and did not satisfactorily show that istradyfilline was effective at reducing the ‘off’ time.^8^ Different interpretations of the value of efficacy data depend on several aspects, including the primary endpoints chosen, condition of the population enrolled into the studies, available and selected comparators and other methodological aspects. EMA and FDA have a program to provide parallel scientific advice, and efforts have been made to understand the different methodological approaches and harmonize them when possible.

Levodopa is still considered the gold standard for the treatment of motor symptoms in patients with PD. As time goes by however, patients treated with levodopa may experience motor fluctuations. Therefore, many efforts were made to develop levodopa formulations with a modified release/different route of administration to avoid fluctuations of levodopa concentrations in the blood and minimize motor fluctuations. Moreover, levodopa-induced dyskinesias and dystonias constitute a major safety concern.^9^ Several controlled studies showed that 16-h continuous administration of continuous intra-jejunal infusion of levodopa-carbidopa intestinal gel (LCIG) results in reduction in motor fluctuations and amelioration of non-motor symptoms in subjects with advanced PD with severe motor fluctuations.^10,11^ LCIG was approved by the FDA in 2015 and Japan in 2016 for the treatment of motor fluctuations in patients with advanced PD.^12^ LCIG is also nationally approved in the EU MS. Moreover, there are several extended or prolonged-release medicinal products containing levodopa-carbidopa available in the EU.^13^ Interestingly, one extended release formulation (Numient) has been centrally approved in EU in 2015 but was never marketed, while its US counterpart (Rytary) has.

In 2019 levodopa in pharmaceutical form of inhalation powder, hard capsule (Inbrija), indicated for the intermittent treatment of OFF episodes in adult PD patients treated with a levodopa/dopa-decarboxylase inhibitor was approved by the EMA.^14^ Inbrija is also approved in the US with a similar indication.^15^

Pimavanserin has been approved in the US since 2016 for the treatment of hallucinations and delusions associated with psychosis experienced by patients with PD.^16^ Pimavanserin is not available in the EU.

Although there are several treatments available for patients with PD, there is an unmet medical need, both for motor (i.e., severe off, falls) or non-motor symptoms, (i.e., cognitive decline, anhedonia). Therefore, further studies, targeting new mechanisms and hopefully acting as a disease modifying treatments are strongly encouraged. Moreover, longitudinal studies are needed to facilitate development of new treatments of non-motor symptoms in patients with PD.

## The regulatory aspects of medicinal products approval: The example of the European medicines agency

EMA provides regulatory and scientific support academics, clinical researchers, non-profit research organizations in the EU as well as drug developers to facilitate development of medicines.^17^ Specifically, the EMA provides Scientific Advice (SA) through the lifecycle of the product on quality, non-clinical and clinical aspects of development to generate robust and adequate evidence for a future marketing authorization, and after marketing, in the development of new indications, to cope with post-approval commitments or emerging safety issues.^18^

The SA is provided by the EMA's Committee for Medicinal Products for Human Use (CHMP) following the recommendation of the Scientific Advice Working Party and, if required other EMA Committees and Working Parties (e.g., Biologics Working Party, Quality Working Party, Committee for Advanced Therapies, Paediatric Committee and the Committee for Orphan Medicinal Products).

During the SA procedure various aspects of development can be discussed with the regulators, including manufacturing, in vitro and in vivo non-clinical tests, aimed to address both the activity and toxicity of the investigational product. When the product reaches clinical phase, patient representatives are also brought in, to share their views. Moreover, clinical aspects, such as overall clinical development program, adequacy of the proposed study population, choice of the does to be evaluated, selection of endpoints, duration of clinical studies, statistical methods, size of the proposed safety database and risk-management plans can be discussed during SA. When approaching MAA, questions on the overall submission for approval strategy, including questions on the adequacy of the proposed datapackge to support a conditional marketing authorization can be raised for discussion in a SA.^19^ The Applicants have an opportunity to meet with the regulators during a Discussion Meeting to present, explain and justify their proposed development program before responses to the questions included in the SA are provided as a Final Advice Letter.

Other EMA platforms support development of medicinal products: Innovation Task Force (ITF), who allows an early dialogue for innovative therapies developments or PRIority MEdicines (PRIME) scheme, dedicated to provide enhanced regulatory support for the development of medicines that target unmet medical needs and have shown promising initial results are examples of those.^20^ Moreover, qualification of novel methodologies (novel biomarkers or imaging methods) can be obtained from the EMA within the remit of Qualification Procedure. Qualification process can lead to either a CHMP Qualification opinion or a qualification advice.^21^ Fee waivers and reductions are included in the EU legislation to facilitate medicine development for small and medium-sized enterprises (SMEs). An ad-hoc executive fee reductions or waivers under a specific legal provision (article 9 of Council Regulation (EC) no. 297/95) can also be provided by the EMA when justified for public health reasons.^22^

EMA disease-specific guidelines are available and are regularly updated – the Guideline on clinical investigation of medicinal products in the treatment of PD (EMA/CHMP/330418/2012 rev. 2) is currently under revision.^23^

## Clinical aspects of development of medicinal products intended for the treatment of PD

Studying PD (or other form of parkinsonism) has always been challenging. The long duration and slow progression of the disease, the heterogeneity of phenomenology and its cyclic nature over the time, be it a day or a month; the impact of effective medication and polytherapy, all are difficult variables to tackle. In addition, correct diagnosis, co-morbidity and iatrogenesis add to the heterogeneity of the patient population.

### Objectives of the clinical trial

The target of the treatment influences the type of design. Traditionally, studies have been designed to cover either symptomatic relief or modification of disease progression. This dichotomy is frequently difficult to achieve. Many tools used to identify disease progression are based upon phenomenology (i.e., MDS-UPDRS) mixing up both contexts. Furthermore, when treatment is required in naïve patients or when dose adjustments are needed on previous stable treated patients, identifying disease progression becomes more difficult. On the other hand, treatment adjustment may not only depend on disease progression, but on the individual needs of the patient, especially if in young and employed. The earlier the position in the natural history of the disease, the more difficult it becomes to control and follow different variables. Ideally, duration of studies to decrease disease progression are in the order of several years, and this has been done in the past.^24^ Given the unmet medical need of certain conditions, and the continuously updated available treatment options, shortening strategies are implemented. The impact that these strategies have on study validity can still not be adequately estimated.

### Study design

When designing a study in PD, the type of study will depend upon the product and the intended indication. If the product has a new mode of action and there is no other product approved for the same indication, then a superiority study (as compared to placebo) is expected. Comparison to placebo facilitates identification of the magnitude of effect of the product and provides assay sensitivity to the study, i.e., that the population enrolled really behaves as expected. A placebo-controlled trial can be carried on a stable treated population. On the other hand, when the product has already similar products approved for the same indication, a non-inferiority trial against an active comparator may provide an easier demonstration of efficacy, while also providing information on the therapeutic added value as compared to the active comparator. This, however, requires identification of the non-inferiority margin, which can be difficult with some endpoints.

The conduct of the study will also depend upon the objective of the study. If the objective is to demonstrate a modification of the natural history of disease progression, a time-to-event approach, where the individual patient is studied until a certain event occurs (e.g., gait disturbance with falls; need to increase baseline medication) can be used. However, this requires a clear definition of the event (e.g., the second unexplained fall when on medication; worsening no longer acceptable requiring dose increase of levodopa), and may hamper safety analysis if the occurrence of events is not distributed along the time during the study. More frequently, a longitudinal, blinded controlled study is chosen.

Identification of expected intercurrent events and the way to deal with them when interpreting the results is also very important. The choice of the primary estimand policy and other supportive strategies should be part of the approved statistical analysis plan (SAP) before results become available.^25^

### Study population

Diagnosis of PD still relies in clinical characteristics. Several attempts to develop minimally or non-invasive diagnostic biomarkers of the disease have long been sought and failed up to now. The biomarker concept merits a deep discussion beyond the remit of this manuscript, as—for the same condition—diagnostic markers frequently differ from disease progression markers or treatment response markers.^26^ Still, biomarker-based diagnostics have been accepted in neurology inflammatory disorders for years (2017 revised Mc Donald criteria in revision - 2024 ECTRIMS update), and as secondary endpoints, as markers of disease progression (burden of T1 black holes, Gd + enhancing lesions).^27^

Conceptually, when the goal is to delay or revert progression of a neurodegenerative disease such as PD, the best strategy would be to develop a safe and efficacious product that could be administered as soon as the diagnosis is made. And the earlier the diagnosis and start of treatment, the best. To facilitate this approach, two new biological frameworks have been developed: the neuronal α-synuclein disease integrated staging system (NSD-ISS)2 and the SynNeurGe3 criteria. There is an ongoing discussion on the objectives and differences, but to the regulator two important questions emerge and require a response at the time of benefit-risk assessment. The first is how can the population who will develop clinical signs of disease and impairment be discriminated from those asymptomatic that will not develop disease within a reasonable timeframe? The second is how does the presymptomatic disease stages progress in time, and when does the staging progression result in identification of symptoms or impairment? This is essential for the benefit-risk assessment in the asymptomatic population and in the following stages.

Population enrichment, by selecting patient characteristics that will facilitate discrimination between treatment arms, is a common strategy to shorten trial duration and sample size. For instance, selecting asymptomatic patients who are on the verge of developing symptoms can be a way to achieve trial results in a shorter timeframe. In fact, enrichment is quite common in the early phases of development (proof of concept, dose finding). Other tools can be used, such as modelling and simulation, and population borrowing from other trials or registries. However, in confirmatory trials, if any of these strategies are used, there is another side of the coin: either the results are easily extrapolated to the general population in the initially planned indication, or the final approved indication may be limited to the tested population.

Presently available medication can provide good quality of life of PD patients for some time. However, most patients develop uncontrollable signs and symptoms, either due to disease progression or to adverse reactions to treatment. Being at an unmet medical need, this population can be especially challenging, due to its variability, co-morbidity and co-medication. Furthermore, some patients may be partly dependent, both motor and cognitively, requiring the input of a caregiver. Trials in these conditions can be difficult to produce and must be carefully designed to optimize the chances of success.

### Study endpoints

The choice of tools to identify the benefit and the risks of a treatment strategy is as much relevant as the study population. The hierarchical choice of the endpoints is very important. While the primary endpoint makes a trial result positive or negative, the secondary endpoints have become increasingly important in regulatory decisions. Key secondary endpoints, which immediately follow the primary endpoint, can also be considered for the statistical power of the study; and, if expressing similar results as the primary endpoint, turn results more robust. Other secondary endpoints, if pointing in the same direction, can provide further support of efficacy (totality of evidence approach). As stated before, the SAP should be cautiously prepared upfront to decrease the risks of a failed study due to lack of statistical significance in clinically relevant endpoints. The clinical and patient relevance of the chosen endpoints is critical to the assessment of benefit. The relevance should be clearly known: the MID (minimal important difference or the minimal change in the scale the is clinically relevant) should already be established and, whenever possible anchored on other known scales.^28^

The move towards patient reported outcome (PRO) has occurred in PD, as well as in other disorders, and is welcomed. However, it is well known that PROs often show a higher placebo effect than clinician outcome assessments (COA) and have not been favored by either sponsors nor regulators for conditions with a high placebo effect such as pain, even in diseases usually considered less prone to placebo effect.^29^ One approach can be to increase sample size and prolong study duration so that the placebo effect slowly wanes, but this is not frequently feasible. Another approach is to improve COAs by accommodating the patient most relevant outcomes.^30^ In this respect, one common mistake identified in the development of new products, is the decision of the sponsor to use selected items of the well-established COA (e.g., MDS-UPDRS part III without tremor related or without rigidity related items), based on the results of phase 2 studies or convenience (virtual visits unable to assess muscle tonus). Most of the times, individual items differences between study arms occur by chance, and this has led to failure of confirmatory clinical trials. This said, improvement of the widely used tools is welcomed, not only to investigators and sponsors, but also to regulators.^31^ To this extent, several consortiums of different stakeholders, including both pharmaceutical industry, academicians, clinicians, patient's associations and regulators have been established. As a rule, these usually start on a pre-competitive space, where available data is assessed and new tools are planned and tested in a study population with the condition to confirm that the clinimetric properties are the desired ones. Testing requires not only cross-sectional but especially longitudinal studies, that show the behavior of the tool along the time and at different disease stages. The process to establish new validated tools is not quick. The alternatives, however, (opinion maker Delphi panels, selection of specific items from validated tools that seem to have facial validity, etc.) are much riskier for all.

## Role of the regulators in the development of medicinal products

Regulators have a unique position among health stakeholders: they have access to confidential information that others do not. While pharmaceutical companies do not have unpublished data from competitors, regulators have access to raw data and know the results of clinical trials in depth for many disorders. Other stakeholders such as politicians, third party payers or the general population have only access to published papers, and official regulatory and transparency rules documents.^32^ Regulatory agents have thus both the chance and responsibility to guide developers of new medications to avoid practicing previous mistakes that led to non-efficacy failed trials, and guide and protect prescribers and patients. Of course, the ultimate decision is on study sponsors, and regulatory activity cannot mandate them to conduct or not the trial they want to have. It is acknowledged that Sponsors also have to take on board other aspects which eventually guide their actions, apart from regulator's view. Prophylaxis is better than treatment, but regulators may, to a certain extent, help to overcome faulty designs by the time of MAA assessment, if the data allows. In the CNS field there have been recent examples where an analysis of the secondary endpoints may strengthen the primary endpoint lack of clinical significance or even failure, or when the intended indication had to be refined so that the product benefit/risk profile is positive in the treated population. Still, it is not rare that the Sponsor ultimately decides to carry on a trial which regulators are pretty sure will fail, not due to the lack of efficacy of the product, but due to a faulty study design, which failed to identify the advantage of the product.

## Conclusion

In neurological disorders, PD has been one of the earliest to have a truly efficacious symptomatic treatment. Yet, in the past 25 years, only a few new treatments were made available for the symptomatic treatment, which has not accompanied the twofold increase in PD population. Several unmet medical needs have been identified for the treatment of mid to late-stage disease, and products capable of delaying the disease pathology are welcomed as well both in the earliest and late stages. While many factors may be accounting for the lack of new available treatments in PD, improvement and acceleration of development of new medicinal products is needed. To do this and keep the mandatory high standards of assessment required, both drug developers, patients advocates and clinicians must engage with regulatory agents as early as possible. Knowing the regulatory framework and how to overcome the common obstacles to production and interpretation of robust data, is key to a successful development drug plan and may facilitate market authorization.

**Table:** Take home information

How can regulators facilitate development of new medicines in PD?
Regulators may provide useful guidance on the development of new medications, by providing advice at different phases of development (chemical, non-clinical, clinical and post-marketing (pharmacovigilance).
On clinical grounds, regulators may advice on selection of: the best design to accomplish the purported objective of treatment study population statistical analysis best tools for proof of concept/dose finding studies and final confirmatory studies expected safety data for marketing authorization
Regulators have access to confidential and propriety data like no other health stakeholder, being able to have a broad picture of developing drugs. Some “conservative” approaches may derive from this position.
